# A Prospective Study to Detect Immune Checkpoint Inhibitors Associated With Myocarditis Among Patients Treated for Lung Cancer

**DOI:** 10.3389/fcvm.2022.878211

**Published:** 2022-06-06

**Authors:** Clara Faubry, Maxime Faure, Anne-Claire Toublanc, Rémi Veillon, Anne-Iris Lemaître, Charlotte Vergnenègre, Hubert Cochet, Sadia Khan, Chantal Raherison, Pierre Dos Santos, Maeva Zysman

**Affiliations:** ^1^Pulmonary Department, Centre Hospitalier Universitaire (CHU) Haut-Lévèque, Bordeaux, France; ^2^Heart Failure Unit, Cardiology Department, Centre Hospitalier Universitaire (CHU) Haut-Lévèque, Bordeaux, France; ^3^Department Cardiology, Lyric Institute, Fondation Bordeaux Université, Bordeaux, France; ^4^Department Medicine, Universitaire Bordeaux, Institut National de la Santé et de la Recherche Médicale (INSERM), Bordeaux, France; ^5^Department of Cardiovascular Imaging, Centre Hospitalier Universitaire (CHU) Bordeaux, Bordeaux, France; ^6^Bordeaux University, Institut National de la Santé et de la Recherche Médicale (INSERM), Bordeaux Population Health Research Center, Bordeaux, France; ^7^Centre de Recherche Cardio-Thoracique, Universitaire Bordeaux, Institut National de la Santé et de la Recherche Médicale (INSERM), Bordeaux, France

**Keywords:** myocarditis, screening, immune checkpoint inhibitors, lung cancer, early diagnosis

## Abstract

**Background:**

Immune checkpoint inhibitors (ICIs) are widely used in lung cancer management. However, myocarditis, which is a rare, yet potentially severe adverse-related event associated with ICIs, could be under-reported.

**Objectives:**

This study is aimed to prospectively evaluate the cumulative incidence rate of myocarditis, through systematic screening, among patients receiving ICIs for lung cancer.

**Methods:**

All patients who received the first administration of ICIs for non-small cell (NSCLC) and small cell lung cancer (SCLC), between May and November 2020, in the pulmonary department of Bordeaux University Hospital, were included. Echocardiography (ECG), troponin-I, and natriuretic peptide dosages before ICIs' first administration and before each infusion were recorded. ECG and magnetic resonance imaging (MRI) were done additionally, in case of at least three times increase in troponin levels, ECG modifications, and the onset of cardiovascular symptoms. Second, if possible, coronarography than endomyocardial biopsy was assessed. The primary outcome was defined as ICIs related to myocarditis onset, while secondary outcomes included other cardiovascular events, disease-free, and overall survival.

**Results:**

During the period of interest, 99 patients received their first infusion of ICIs for lung cancer (mean age 64 ± 9 years; 52 men, 67% with adenocarcinoma). Three cases of myocarditis without major adverse cardiac events (MACEs) occurred (two definite and one possible), and the mean duration between the first ICIs' administration and myocarditis onset was 144 ± 3 days. Median disease-free survival and overall survival were 169 [102; 233] days and 209 [147; 249] days, respectively.

**Conclusion:**

In our study, systematic screening of myocarditis associated with ICIs leads to a more frequent incidence and a later onset than previously reported. None of them were severe. Additional prospective evidence is needed before we could adopt routine cardiac screening in unselected patients starting ICIs; however, these data shed new light on the risk of myocarditis associated with ICIs administration.

## Introduction

Immune checkpoint inhibitors (ICIs) have substantially improved clinical outcomes in multiple cancer types, such as lung cancer (1). In France, there are currently four approved ICIs in lung cancer, which are as follows: nivolumab, pembrolizumab, (both anti-programmed death-1, PD-1), atezolizumab, and durvalumab (both anti-programmed death ligand-1, PD-L1). The indications for their use in lung cancer treatment continue to expand and are often considered the first-line therapy (2). Unfortunately, these agents may induce a wide spectrum of immune-related adverse events (irAEs) by enhancing immune responses in non-target organs (3), including the cardiovascular system. While myocarditis is considered uncommon toxicity of ICIs with incidence varying from 0.01 to 1% (2). However, it is likely that myocarditis is under-reported, owing to an absence of systematic monitoring and coding mechanisms for cardiac events in immunotherapy trials. Moreover, myocarditis related to ICIs has been described to have a fulminant course, with a fatality rate of 30–50% (2). A meta-analysis of the incidence of immune-related adverse effects in patients treated for advanced non-small cell lung cancer (NSCLC) identified that myocarditis affects 0.5% of the whole population (3). Additionally, myocarditis has been reported to be differentially associated with available ICIs. For example, a combination of pembrolizumab and ipilimumab has shown a higher reporting of myocarditis as compared to one ICI alone or in combination with chemotherapy (4, 5). Although, myocarditis can also occur with immunotherapy administered alone (6). IrAEs may occur secondary to the inhibition of immune checkpoints leading to local and systemic auto-immune responses (CD4+ and CD8+ T cells recruitment along with macrophages infiltrate), which attack myocytes and cardiac conduction tissue that cause myocarditis (7).

Until now, the reported median time of the onset of myocarditis from the first ICIs' infusion ranges from 34 [21; 75] to 65 [2; 454] days (8). Since cardiac monitoring (e.g., ECG or troponin) is not routinely performed, in most immunotherapy trials or clinical practice, the true incidence of myocarditis remains still unknown. The diagnosis of myocarditis can be based on appropriate investigations as recommended by the European Society of Cardiology Guidelines (9). Interestingly, the clinical presentation of myocarditis ranges on a spectrum of mild-to-severe diseases from an asymptomatic increment in cardiac biomarkers to severe decompensation with end-organ damage, as suggested by clinical practice guidelines for the management of irAEs (10). Therefore, the need for increasing awareness to suspect, diagnose, and treat ICI-related myocarditis is pivotal in lung cancer patients who receive ICI treatment.

Hence, the aim of this study was to prospectively evaluate (1) the incidence of myocarditis associated with ICIs administration and (2) the frequency of other major cardiovascular events, such as ischemic heart disease or heart failure, in stages IIIB–IV lung cancer patients.

## Materials and Methods

### Study Design

All adult patients who initiated ICI treatment for stages IIIB–IV lung cancer between 1 May 2020 and 1 November 2020, in the pulmonary department of Bordeaux University Hospital, were included. All participants provided informed written consent. All patients who did not receive the first administration of ICI were excluded.

### Sample Size

The cumulative incidence rate of myocarditis associated with ICIs' administration varies from 0.01 to 1% (2). The hypothesis is that the event is under-reported. Based on previous work, we had anticipated a cumulative incidence rate of 2%. For an α-error of 5% and a β-error of 10%, the number of patients required was 92. In order to take into account missing data or withdrawals of consent, a total of 98 patients needed to be included.

### Ethical Approval and Consent to Participate

The study protocol was approved by the Ethics Committee of CHU Bordeaux (France) and registered with the following number CHUBX2020RE0275. This work complies with the protection of personal health data and the protection of privacy with the framework of application provided by article 65-2 of the amended Data Protection Act and the general data protection regulations. All subjects provided informed written consent. All authors provided consent to publication.

### Data Collected

The following data were collected: demographic characteristics, smoking history, pre-existing cardiovascular diseases (coronary artery disease, arrhythmia, conduction abnormalities, and heart failure), lung cancer type [NSCLC and small lung cancer (SCLC)], grading [stages IIIB and IV, according to the 7th American Joint Comission on Cancer Tumor Node Metastasis (AJCC TNM) classification], ICI regimens, a combination of ICIs and chemotherapy, number of lines, pre-existing auto-immune diseases, and other immune side effects during treatment. We used The Strengthening the Reporting of Observational studies in Epidemiology (STROBE) reporting guidelines in our study (Supplementary Data 1).

### Myocarditis Suspicion

Baseline troponin and natriuretic peptide levels, ECG, and trans-thoracic echocardiography (TTE) were performed before the first ICI infusion to evaluate possible changes from baseline, e.g., changes in left ventricular ejection fraction (LVEF), diastolic function, new wall motion abnormalities, or pericardial effusion. Prior to ICI's administration, levels of biomarkers (troponin-I and natriuretic peptide) and ECG measurements were undertaken. Possible myocarditis was suspected, in case of any 1 of the following adverse events: new cardiovascular symptoms or at least 3 times increase in the levels of biomarkers beyond the level prior to ICI's administration, or any of the following ECG changes: new prolongation of the PR interval, atrioventricular block, ventricular arrhythmias, frequent premature ventricular complexes, ST depression, or diffuse T-wave inversions.

In the presence of an adverse event, additional scans were performed, which are as follows: TTE, cardiac magnetic resonance imaging (C-MRI), and coronarography. C-MRI was assessed with T2-weighted imaging, late gadolinium enhancement (LGE), extracellular volume fraction, and T1 and T2 mapping.

For the C-MRI diagnosis of myocarditis, the Lake Louise Criteria were used in our study, which states that if (2, 11) both myocardial edema and non-ischemic myocardial injury are identified on the C-MRI, it is highly suggestive of myocarditis.

### Myocarditis Diagnosis

Any one of the following criteria is used to diagnose myocarditis in a clinical setting, the presence of two major criteria having the best diagnostic value:

a. Myocardial edema: Indicated by abnormal findings in T2 mapping or T2-weighted images.
b. Non-ischemic myocardial injury: Ascertained by abnormal findings on T1 mapping, LGE, or extracellular volume fraction.

Additional supportive criteria (below) can be suggestive of myocarditis, however, in the absence of the aforementioned two criteria, they cannot be considered definitively diagnostic of myocarditis.

a. Pericarditis: Indicated by either pericardial effusion or abnormal late gadolinium enhancement/T2 or T1 findings in the pericardium.
b. Left ventricular systolic dysfunction: Indicated by regional or global wall motion abnormalities.

Coronary angiography was performed to rule out significant coronary artery disease. Then, endomyocardial biopsies were performed when possible and guided according to C-MRI abnormalities. The myocardial tissue was evaluated using the histological Dallas criteria, which require two main components: inflammatory infiltrate and myocardial necrosis (12). If myocarditis was suspected, it was categorized as definite/probable/possible per consensus-based definition (13). Finally, treatments for myocarditis were decided according to international recommendations (2).

### Statistical Analysis

Data are provided as mean or *n* (%), as appropriate. A value of *p* ≤ 0.05 was considered statistically significant. All analyses were performed using Graph Pad Prism® statistical software.

## Results

**Figure 1:**
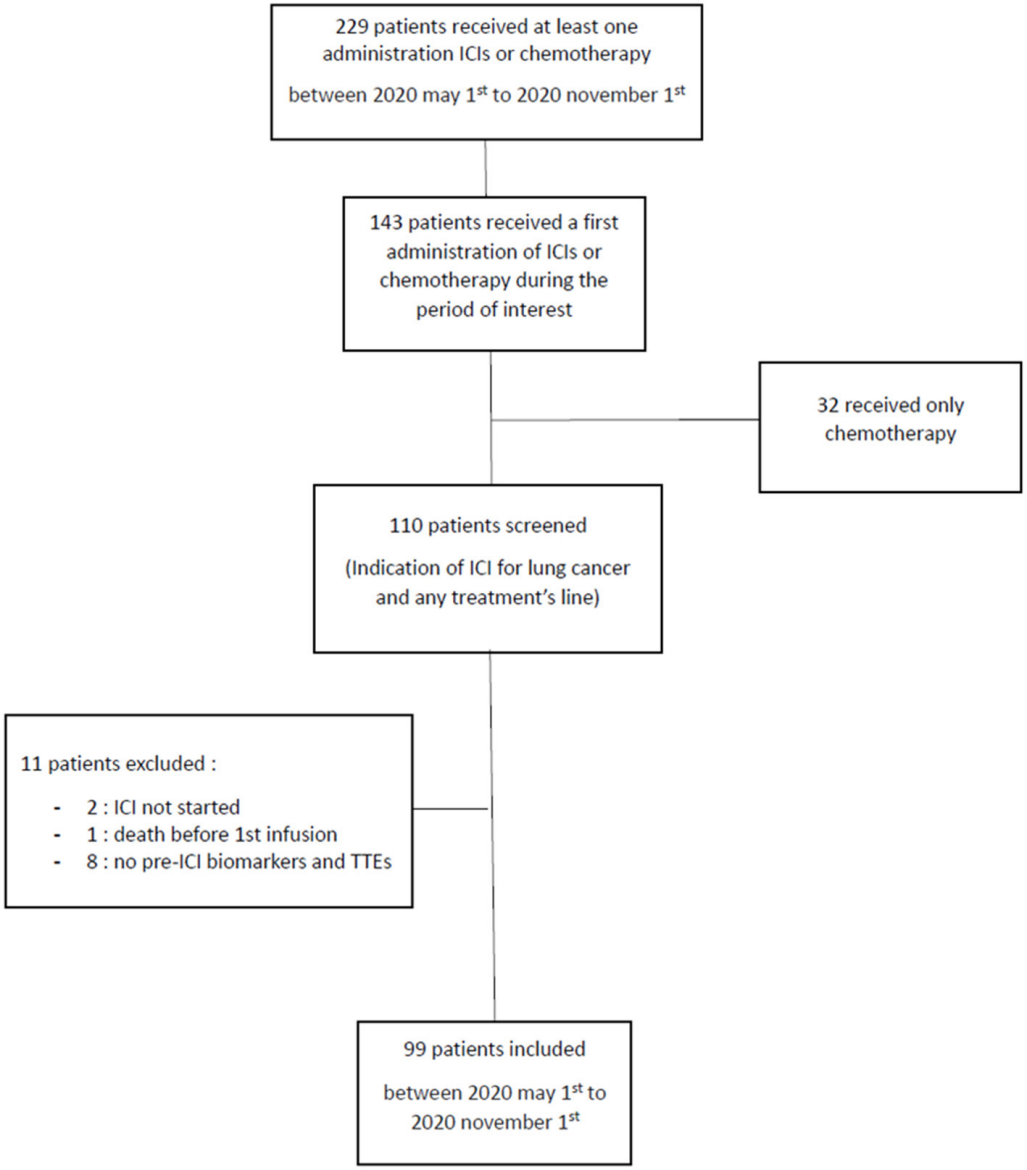
Flow chart. ICI, immune checkpoint inhibitors; TTE, transthoracic echocardiogram.

Between 1 May 2020 and 1 November 2020, 99 patients (52% men, mean age: 64 ± 9 years) received the first administration of ICIs (Figure 1). In total, 38% of patients had pre-existing cardiovascular diseases and 15% suffered from pre-existing systolic or diastolic dysfunction. In addition, 6% of patients had positive troponin before starting treatment among whom, two patients had pre-existing stable ischemic heart disease and 1 presented tight aortic stenosis. The majority of the patients (66%) had adenocarcinoma (Table 1) and 67% were being treated with a combination of ICIs and chemotherapy, while 61% received first-line treatment. In total, 72% of patients received Pembrolizumab (Table 2).

**Table 1:** Patients' characteristics at inclusion.

	**Patients' characteristics**	**Whole cohort**
		***N* = 99 (%)**
General		
	Mean age (years)	64
	Male gender	51 (52%)
Risk factor	Smoking	
	No	10 (10%)
	Cessation >3years	40 (40%)
	Current	49 (49%)
	Preexisting cardiovascular diseases	
	Coronary artery disease	14 (14%)
	Other artery disease	11 (11%)
	Arrhythmia/Conduction abnormality	13 (13%)
	Heart failure	
	LVEF <40%	1 (1%)
	LVEF 40–50%	6(6%)
	LVEF >50%	8(8%)
	Cardiovascular risk factors	
	Age (male >50 years; female >60 years)	80 (81%)
	Diabetes mellitus	19 (19%)
	Dyslipidaemia	32 (32%)
	Hypertension	18 (18%)
Primary cancer type	Adenocarcinoma	65 (66%)
	Squamous cell carcinoma	17 (17%)
	Small cell lung cancer	12 (12%)
	Others	5 (5%)
Pre-ICI biomarkers	Positive serum troponin before ICI	6 (6%)
	Troponin (ng/l)	13+/−21^μ^
	BNP (pg/ml)	44+/−41^π^
	NT-pro-BNP (pg/ml)	341+/−448^Ω^
	CPK (UI/l)	64+/−55^†^
Pre-ICI ECG	*PR (ms)*	153+/−27^‡^
	*QRS (ms)*	94+/−21^ 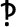 ^
Pre-ICI TTE	*LVEG (%)*	61%+/−6.5%^ 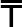 ^
	*Strain (%)*	−18%+/−3.1%^ 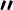 ^
	*S'VD (cm/s)*	13.6+/−2.66^ 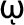 ^

*Missing data :*

μ *= 8 (8%)*,

π *= 25 (25%)*,

† *= 14 (14%)*,

‡ *= 11 (11%)*,

*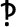 =9 (9%), 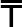 = 2 (2%), 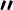 = 39 (39%), 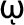 = 14 (14%)Patients concerned :*

Ω *= 15 (15%)*.

*ECG, electrocardiogram; ICI, Immune checkpoint inhibitors; TTE, Transthoracic echocardiogram; LVEG, Left ventricular ejection fraction*.

*Data were expressed as mean +/- standard deviation, as appropriate*.

**Table 2:** Patients' follow-up.

		**Whole cohort**
		***N* = 99(%)**
ICI regimens	Pembrolizumab	71 (72%)
	Nivolumab	7 (7%)
	Atezolizumab	8 (8%)
	Durvalumab	11 (11%)
	Other	2 (2%)
Single agent or combined	Monotherapy	33 (33%)
	Combinaison	66 (67%)
Line of treatment	1st line	60 (61%)
	2nd line	33 (33%)
	≥3rd line	6 (6%)
		
Myocarditis		3 (3%)
Follow-up	Median follow-up (days)	209 [147; 249]
	Mortality rate	28 (28%)

*ICI, Immune checkpoint inhibitors*.

Myocarditis was diagnosed in three patients during the 6-month follow-up (two definite and one possible, Table 3), indicating a cumulative incidence rate of 3%. All of them were asymptomatic. Troponin serum increment was seen for all three patients: 75.0 ng/l in the first case, 20.8 ng/l in the second case, and 202.0 ng/l in the third case. None of them had elevated troponin levels prior to ICIs. The mean duration between the first ICI administration and the onset of myocarditis was 144 ± 3 days (147 days for the first and the second cases; 141 days for the third case). Grade 1 skin toxicity (irAE) was seen between the first and second infusions for one patient with myocarditis (patient 2); however, no pre-existing auto-immune disease was previously reported (Table 3). No ECG abnormalities were seen, and TTE revealed preserved LVEF for all patients. We were able to distinguish myocarditis from myocardial ischemia or myocardial infarction with early systematic coronary angiography.

**Table 3:** Description of myocarditis cases.

	**1st patient**	**2nd patient**	**3rd patient**
Primary cancer type	Squamous cell carcinoma	Adenocarcinoma	Small cell lung cancer
ICI regimens	Atezolizumab	Pembrolizumab	Atezolizumab
Single agent or combined	Monotherapy	Combinaison	Combinaison
Line of treatment	2nd line	1st line	1st line
Pre-existing auto-immune diseases	No	No	No
Other immune side effects during treatment	No	Dermatitis (grade I)	no
Time from first administration to myocarditis (days)	147	147	141
Biomarkers			
Serum troponin (ng/l) standard <15,6 (ng/l)	75	20,8	202
BNP (pg/ml)	13	21	45
CPK (UI/l)	38	57	33
ECG			
Sinus rythm	Yes	Yes	Yes
PR (ms)	160	178	160
QRS (ms)	100	96	80
TTE			
LVEF(%)	53	61	65
Strain(%)	−19.5	Not performed	Not performed
S'VD (cm/s)	11.5	Not performed	Not performed
Cardiac-MRI			
Edema by T2	Yes	No	Yes
Late Gadolinium enhancement	Yes	No	Yes
Coronary angiography	negative	Negative	Negative
Endomyocardial biopsy	Non specific edema	Not performed	Not performed
Final diagnosis	Definite myocarditis	Possible myocarditis	Definite myocarditis
ICI rechallenge			
Yes/no	yes	Yes	No
ICI regimen	Nivolumab	Pembrolizumab	-
Time to rechallenge (in days)	124	71	-
Recurrence yes/no	No	Yes	-

*ECG, electrocardiogram; ICI, Immune checkpoint inhibitors; TTE, Transthoracic echocardiogram; LVEG, Left ventricular ejection fraction; Cardiac-MRI, Cardiac Magnetic Resonance Imaging*.

**Figure 2:**
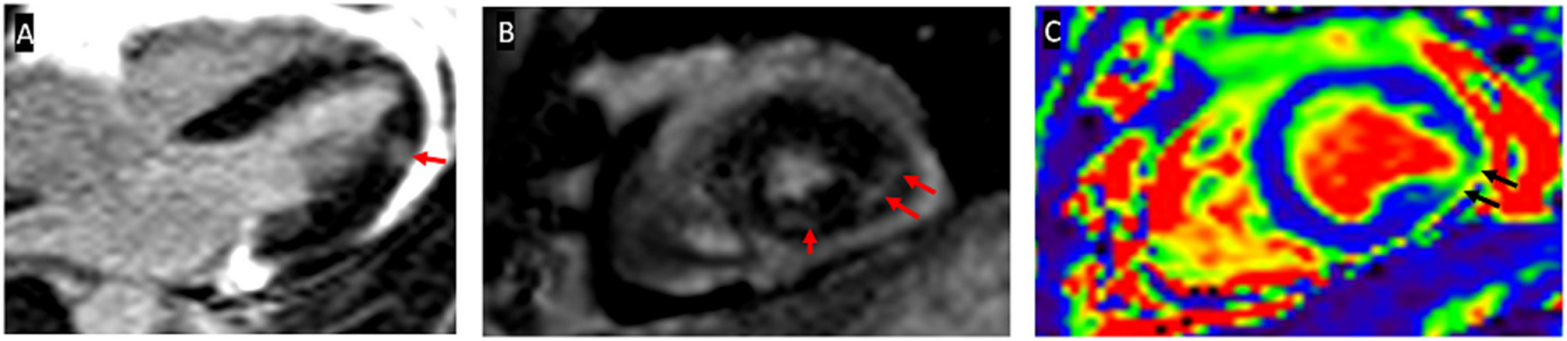
Cardiac magnetic resonance imaging (C-MRI) imaging illustration of patient 1. **(A)** 4 cavity sections with a late enhancement of gadolinium and **(B)** transversal section showing infero-latero-medial mesomyocardic contrast (red arrows). **(C)** T2 mapping showing focal infero-latero-medial myocardial edema (black arrows).

The first patient had an asymptomatic elevation of cardiac biomarkers. C-MRI showed myocardial edema in T2 mapping and LGE in a non-coronary distribution (Figure 2). Endomyocardial biopsies were performed according to C-MRI abnormalities and found non-specific edema. This patient was classified as having definite myocarditis because of increased cardiac biomarkers, positive C-MRI, and negative coronary angiography (13). The second patient was classified as possible myocarditis because of asymptomatic elevation of cardiac biomarkers, with negative C-MRI and angiography for coronary artery disease (13). In addition, the third case was classified as having definite myocarditis because of asymptomatic elevation of cardiac biomarkers, positive C-MRI but negative angiography for coronary artery disease (Table 3) (13).

All three patients received corticosteroids as recommended (10, 14, 15), intravenous methylprednisolone, at a dosage of 1 mg/kg/day for the first patient, and oral prednisone, at a dosage of 1 mg/kg/day, with no additional immunosuppressant drugs for the remaining patients. The treatments administered were in line with the American Society of Clinical Oncology (ASCO) clinical practice guidelines for irAEs, and troponin monitoring was also done (10). All three patients had mild myocarditis and recovered without complications. No major adverse cardiac events (MACEs), e.g., cardiovascular death, cardiac arrest, cardiogenic shock, and hemodynamically significant complete heart block requiring a pacemaker, were noted.

Considering the non-severe presentation and the absence of alternative choice, ICIs rechallenge was performed in the first and second cases, after the normalization of troponin level. In the first case, nivolumab was administered 124 days after myocarditis and continued due to the absence of a recurrence. In the second case, pembrolizumab was re-administrated, 71 days after myocarditis and recurrence occurred 42 days (three infusions) after rechallenge. The recurrence was detected by ECG changes (*de novo* left ventricular block) and serum troponin increment, while C-MRI was normal. An endomyocardial biopsy was not performed because of a negative benefit-risk balance. Diagnosis of myocarditis was retained in view of ECG normalization under corticosteroid. No rechallenge was performed for the third patient.

After a 6-month follow-up, median disease-free survival and overall survival were 169 [102; 233] and 209 [147; 249] days, respectively. Mortality did not increase among patients with myocarditis at the end of follow-up (*p* = 0.29; not shown).

For one patient, due to the increased serum troponin levels, coronary angiography was performed and it confirmed an underlying coronary artery disease, which was treated with angioplasty. The patient had not previously reported pre-existing cardiovascular disease; however, several cardiovascular risk factors (current smoking, age >50 years) were noted. Finally, systematic TTE before ICIs' first administration allowed us to detect eight cases of unknown systolic or diastolic dysfunction, of which only one had LVEF between 40 and 50%, leading to a specific treatment; while the remaining seven had diastolic heart failure. Additionally, concerning the five patients with positive troponin before starting treatment, two patients had known stable ischemic heart disease and one patient had severe unknown aortic stenosis without surgical indication. The two last patients had a spontaneous normalization of troponin levels.

## Discussion

In our prospective, hospital-based real-life cohort study, the screening of myocarditis was systematically assessed in 99 patients with lung cancer (stages IIIB–IV), receiving ICIs treatment. A 3% cumulative incidence rate of myocarditis was seen during a 6-month follow-up. All cases of myocarditis were mild and without MACEs. No increase in mortality was observed among patients with myocarditis. Finally, myocarditis occurred later than described in previous studies, i.e., the mean time of the onset between the first ICIs' administration and myocarditis was 144 ± 3 days.

No specific clinical characteristics were identified with myocarditis onset; the three cases were different, in terms of histological cancer type, ICIs regimens, combination regimen, and line number. None of the cases had an underlying auto-immune disease. The first case had a history of coronary artery disease without heart failure and the second had a history of valve disease, without associated heart failure.

In addition, the incidence of myocarditis (3%) was higher than previously reported, range of 0.06–1.14% (2), which could perhaps be explained by the rigorous systematic monitoring and subsequent early detection of myocarditis. Systematic monitoring to detect myocarditis is not routinely performed in patients receiving ICI; which might lead to its under-reporting. While the incidence of myocarditis was high (3%); however, the cases were mild and did not affect mortality. This finding was contrary to the previous reporting of a fatality rate of 30–50%, associated with myocarditis due to ICIs treatment (2). The lack of myocarditis-related mortality in our study could perhaps be due to the compliance of the patients with monotherapy, e.g., a large safety database suggests that myocarditis occurs more frequently and severely with the combination of ipilimumab and nivolumab when compared to monotherapy (5). Another hypothesis could be that systematic screening leads to earlier detection of myocarditis. This in turn allowed a prompt withdrawal of ICIs and initiation of corticosteroid treatment (intra-venous or oral) to avoid a fulminant course (16). Moreover, myocarditis had a later onset than previously observed in other studies (8, 16), which further underscores the need for prompt and rigorous systematic detection.

We also noticed a trend of better survival among patients who had myocarditis, suggesting a strong immune response. These results correspond to the findings in the meta-analysis from Hussaini et al. (17) which state that immunotherapy has better efficacy in patients who developed irAEs in different cancers, such as lung cancer.

Besides, after troponin normalization under corticosteroid therapy and in the absence of therapeutic alternatives, rechallenge (8, 10) was considered in the first two cases, with a recurrence of mild myocarditis for the second case, but not for the first one.

Interestingly, all myocarditis presented a normal LVEF in TTEs. C-MRI was normal for one of the cases, and an endomyocardial biopsy was performed only once. Normal results are frequently seen in the early phase of the disease (7), with normal C-MRI being reported in almost 70% of patients (2). In conclusion, ICI-related myocarditis is a complex disease that bears resemblance to many other acute cardiac syndromes. Its diagnosis is difficult as it is based on a combination of different non-pathognomonic parameters, such as biomarkers (troponin, natriuretic, peptides), imaging (ECG, TTE, and C-MRI), and procedures (endomyocardial biopsy and coronary angiography). However, given the high incidence (3%) observed in this study, it is recommended to perform systemic screenings until more definitive data become available (18). C-MRI and endomyocardial biopsies are not available in all medical centers and due to their invasive nature might be unsuitable for asymptomatic patients. Our study indicates that while TTE does not help in the early diagnosis of myocarditis, it is relevant for screening other cardiovascular events. In fact, pre-therapeutic TTE detected 8 cases of heart failure and 3 cases of valve diseases. Of note, even if the first manifestations of myocarditis can be serious cardiac complications, e.g., ventricular arrhythmias and atrioventricular block, the LVEF is often preserved (5, 8, 16). For example, in a study by Mahmood et al. (18) 51% of patients with ICIs associated with myocarditis had a normal LVEF. In addition, in 38% of myocarditis patients, a normal LVEF was seen despite the development of MACEs.

Finally, smokers are at risk of lung cancer and atherosclerosis (19) making them a particularly vulnerable population for MACEs. In a large study, 66% of patients with cancer (*n* = 60,676) also presented with an acute coronary syndrome; and the most prevalent cancers were lymphoma (19%) and lung cancer (18.3%) (20). Contrastingly, in a more specific study by van-Herk-Sukel et al. (21) patients with lung cancer (*N* = 3,717) did not show a higher risk of developing myocardial infarction when compared with cancer-free controls. In our study, only 1 patient with cardiovascular risk factors had an elevation of troponin level linked to coronary artery disease and died a few months after diagnosis. However, cardiovascular co-morbidities (heart failure, myocardial infarction, and cardiac arrhythmias), which have been seen with low survival, in a study of 95,167 lung cancer patients, must be detected as earliest as possible (22).

### Strengths and Clinical Perspectives

To our knowledge, this is the largest published prospective study of ICI-associated myocarditis among patients with lung cancer. While no specific clinical characteristics were identified with myocarditis onset, our study does outline the advantages of using an early and sustained systematic screening strategy for detecting myocarditis, when treating lung cancer patients with ICIs. The rigorous screening allowed for the early diagnosis and management of three cases of mild myocarditis and by extension could lead to a reduction in mortality.

### Study Limitations

This study has several important limitations, such as the small number of patients; therefore, we could not compare overall survival and progression-free survival depending on myocarditis occurrence. Our study was also monocentric with a possible center effect, in particular, for C-MRI and endomyocardial biopsy access. While the probability of an over-diagnosis should be considered with any screening test; however, in our study there was only 1 troponin increment (leading to angioplasty).

## Conclusion

This study outlines the usefulness of early monitoring for myocarditis in patients with lung cancer being treated with ICIs. Early monitoring is especially helpful in cases with non-specific symptoms and would help in decreasing the risk of fulminant progression of myocarditis. However, larger patient cohorts will be needed to estimate the true incidence of clinically meaningful immune-related cardiac events/myocarditis and importantly evaluate potential predictors to define higher-risk subgroups and refine screening and management strategies. Although improved detection and management of immune-related cardiovascular events are important, additional prospective evidence is needed before we can adopt routine cardiac screening in unselected patients starting ICI therapy.

## Data Availability Statement

The raw data supporting the conclusions of this article will be made available by the authors, without undue reservation.

## Ethics Statement

The studies involving human participants were reviewed and approved by Ethics Committee of CHU Bordeaux (France) CHUBX2020RE0275. The patients/participants provided their written informed consent to participate in this study.

## Author Contributions

CF, MF, A-CT, RV, A-IL, CV, HC, SK, CR, PD, and MZ made substantial contributions to the conception and design, acquisition of data, analysis and interpretation of data, and involved in drafting the manuscript or revising it critically for important intellectual content and have given final approval of the version to be published. All authors contributed to the article and approved the submitted version.

## Conflict of Interest

The authors declare that the research was conducted in the absence of any commercial or financial relationships that could be construed as a potential conflict of interest.

## Publisher's Note

All claims expressed in this article are solely those of the authors and do not necessarily represent those of their affiliated organizations, or those of the publisher, the editors and the reviewers. Any product that may be evaluated in this article, or claim that may be made by its manufacturer, is not guaranteed or endorsed by the publisher.

## Abbreviations

C-MRI: cardiac magnetic resonance imaging
ECG: electrocardiogram
ICIs: immune checkpoint inhibitors
irAEs: immune-related adverse event
TTE: trans-thoracic echocardiography
MACEs: major adverse cardiovascular events.
