# Supplementary material for: A Prospective Study to Detect Immune Checkpoint Inhibitors Associated With Myocarditis Among Patients Treated for Lung Cancer
Source: Front Cardiovasc Med. 2022 Jun 6;9:878211. doi: 10.3389/fcvm.2022.878211 (PMC9207328; doi:10.3389/fcvm.2022.878211)
Supplement: Supplementary file 1 [file Data_Sheet_1.pdf]

STROBE Statement—Checklist of items that should be included in reports of ***cohort studies***

A prospective study to detect immune checkpoint inhibitors' associated Myocarditis, among patients treated for lung cancer

	Item No	Recommendation	Page No
**Title and abstract**	1	(*a*) Indicate the study's design with a commonly used term in the title or the abstract	YES
		(*b*) Provide in the abstract an informative and balanced summary of what was done and what was found	P2 L1-23 abstract
**Introduction**			
Background/rationale	2	Explain the scientific background and rationale for the investigation being reported	P3 L24-25
Objectives	3	State specific objectives, including any prespecified hypotheses	P4 L10-13
**Methods**			
Study design	4	Present key elements of study design early in the paper	P4 L16-17
Setting	5	Describe the setting, locations, and relevant dates, including periods of recruitment, exposure, follow-up, and data collection	P4 L16-21
Participants	6	(*a*) Give the eligibility criteria, and the sources and methods of selection of participants. Describe methods of follow-up	P4 L16-P5 L7
		(*b*) For matched studies, give matching criteria and number of exposed and unexposed	Not relevant
Variables	7	Clearly define all outcomes, exposures, predictors, potential confounders, and effect modifiers. Give diagnostic criteria, if applicable	P5 L7-P6 L3
Data sources/ measurement	8*	For each variable of interest, give sources of data and details of methods of assessment (measurement). Describe comparability of assessment methods if there is more than one group	Not relevant
Bias	9	Describe any efforts to address potential sources of bias	Not relevant
Study size	10	Explain how the study size was arrived at	Not relevant
Quantitative variables	11	Explain how quantitative variables were handled in the analyses. If applicable, describe which groupings were chosen and why	Not relevant
Statistical methods	12	(*a*) Describe all statistical methods, including those used to control for confounding	P6 L5-11
		(*b*) Describe any methods used to examine subgroups and interactions	Not relevant
		(*c*) Explain how missing data were addressed	Not relevant
		(*d*) If applicable, explain how loss to follow-up was addressed	Not relevant
		(*e*) Describe any sensitivity analyses	Not relevant
**Results**			
Participants	13*	(a) Report numbers of individuals at each stage of study—eg numbers potentially eligible, examined for eligibility, confirmed eligible, included in the study, completing follow-up, and analysed	P6 L14-16
		(b) Give reasons for non-participation at each stage	Figure 1
		(c) Consider use of a flow diagram	Figure

			1
Descriptive data	14*	(a) Give characteristics of study participants (eg demographic, clinical, social) and information on exposures and potential confounders	P6 L16-20 Table 1
		(b) Indicate number of participants with missing data for each variable of interest	Table 1
		(c) Summarise follow-up time (eg, average and total amount)	P8 L3
Outcome data	15*	Report numbers of outcome events or summary measures over time	P6 L20

Main results	16	(*a*) Give unadjusted estimates and, if applicable, confounder-adjusted estimates and their precision (eg, 95% confidence interval). Make clear which confounders were adjusted for and why they were included	Not relevant
		(*b*) Report category boundaries when continuous variables were categorized	Not relevant
		(*c*) If relevant, consider translating estimates of relative risk into absolute risk for a meaningful time period	Not relevant
Other analyses	17	Report other analyses done—eg analyses of subgroups and interactions, and sensitivity analyses	Not relevant
**Discussion**			
Key results	18	Summarise key results with reference to study objectives	P8 L16-20
Limitations	19	Discuss limitations of the study, taking into account sources of potential bias or imprecision. Discuss both direction and magnitude of any potential bias	P10 L23-25
Interpretation	20	Give a cautious overall interpretation of results considering objectives, limitations, multiplicity of analyses, results from similar studies, and other relevant evidence	P7 L7-13
Generalisability	21	Discuss the generalisability (external validity) of the study results	P11 L11-13
**Other information**			
Funding	22	Give the source of funding and the role of the funders for the present study and, if applicable, for the original study on which the present article is based	Not relevant

*Give information separately for exposed and unexposed groups.

Note: An Explanation and Elaboration article discusses each checklist item and gives methodological background and published examples of transparent reporting. The STROBE checklist is best used in conjunction with this article (freely available on the Web sites of PLoS Medicine at http://www.plosmedicine.org/, Annals of Internal Medicine at http://www.annals.org/, and Epidemiology at http://www.epidem.com/). Information on the STROBE Initiative is available at http://www.strobe-statement.org.
