# Supplementary figures and images for: A Prospective Study to Detect Immune Checkpoint Inhibitors Associated With Myocarditis Among Patients Treated for Lung Cancer
Source: Front Cardiovasc Med. 2022 Jun 6;9:878211. doi: 10.3389/fcvm.2022.878211 (PMC9207328; doi:10.3389/fcvm.2022.878211)

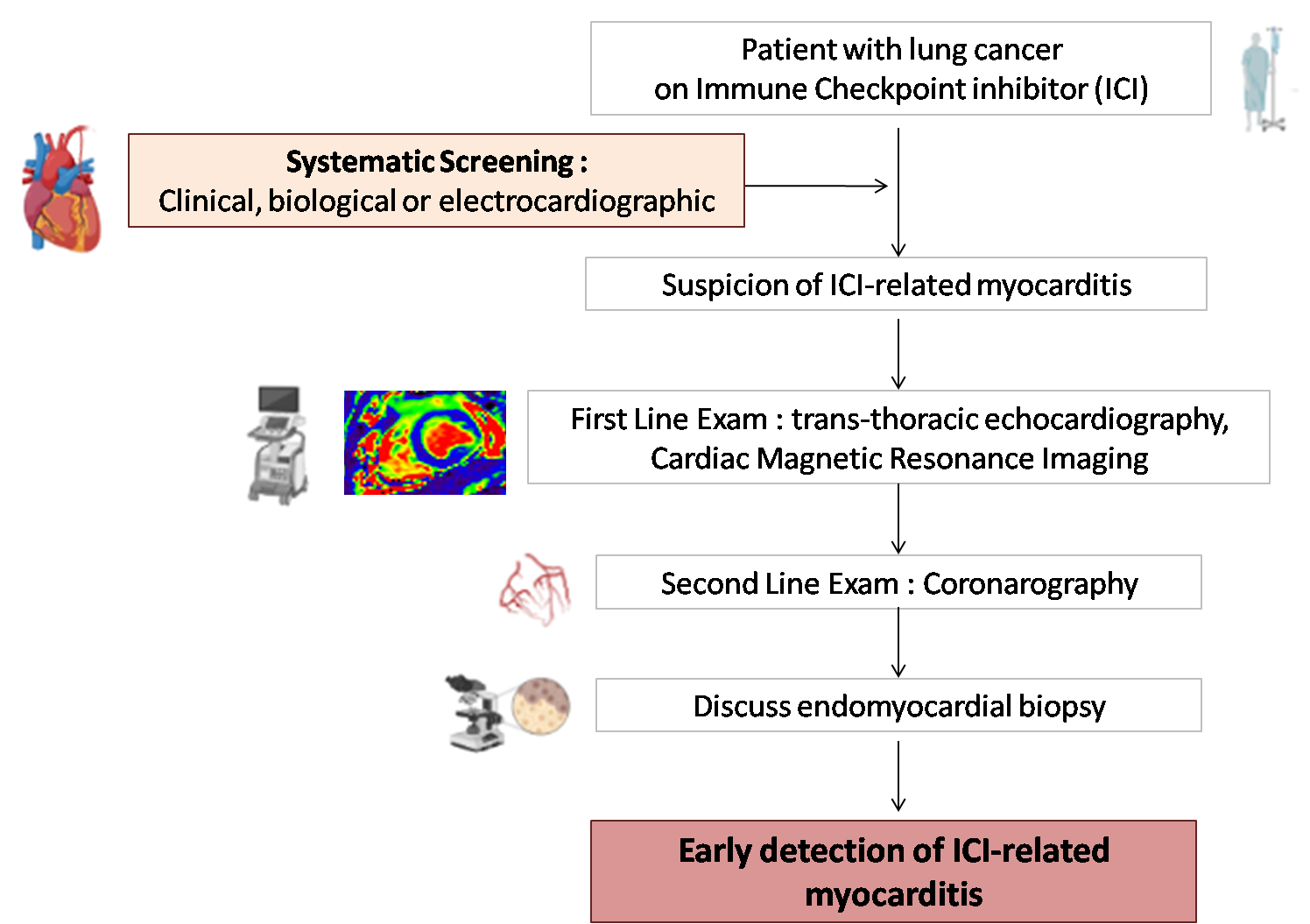

Supplement: Supplementary file 2 [file Image_1.PNG]
